# Linear resistivity at van Hove singularities in twisted bilayer WSe_2_

**DOI:** 10.1073/pnas.2321665121

**Published:** 2024-04-09

**Authors:** LingNan Wei, Qiaoling Xu, Yangchen He, Qingxin Li, Yan Huang, Wang Zhu, Kenji Watanabe, Takashi Taniguchi, Martin Claassen, Daniel A. Rhodes, Dante M. Kennes, Lede Xian, Angel Rubio, Lei Wang

**Affiliations:** ^a^National Laboratory of Solid-State Microstructures, School of Physics, Nanjing University, Nanjing 210093, China; ^b^Songshan Lake Materials Laboratory, Dongguan, Guangdong 523808, China; ^c^College of Physics and Electronic Engineering, Center for Computational Sciences, Sichuan Normal University, Chengdu 610068, China; ^d^Department of Materials Science and Engineering, University of Wisconsin, Madison, WI 53706; ^e^Research Center for Electronic and Optical Materials, National Institute for Materials Science, Tsukuba 305-0044, Japan; ^f^Research Center for Materials Nanoarchitectonics, National Institute for Materials Science, Tsukuba 305-0044, Japan; ^g^Department of Physics and Astronomy, University of Pennsylvania, Philadelphia, PA 19104; ^h^Institut für Theorie der Statistischen Physik, Rheinisch-Westfälische Technische Hochschule Aachen University and Jülich Aachen Research Alliance-Fundamentals of Future Information Technology, Aachen 52056, Germany; ^i^Max Planck Institute for the Structure and Dynamics of Matter, Center for Free-Electron Laser Science, Hamburg 22761, Germany; ^j^Center for Computational Quantum Physics, Simons Foundation Flatiron Institute, New York, NY 10010; ^k^Collaborative Innovation Center of Advanced Microstructures, Nanjing University, Nanjing 210093, China

**Keywords:** non-Fermi liquid behavior, van Hove singularity, twisted bilayer WSe_2_, correlated phenomena, ab initio electronic structure

## Abstract

Different mechanisms driving a linear temperature dependence of the resistivity *ρ* ∼ *T* at van Hove singularities (VHSs) or metal-insulator transitions when doping a Mott insulator are being debated intensively with competing theoretical proposals. We experimentally investigate this using the exceptional tunability of twisted bilayer (TB) WSe_2_ by tracking the parameter regions where linear-in-*T* resistivity is found in dependency of displacement fields, filling, and magnetic fields. We find that even when the VHSs are tuned rather far away from the half-filling point and the Mott insulating transition is absent, the *T*-linear resistivity persists at the VHSs. When doping away from the VHSs, the *T*-linear behavior quickly transitions into a Fermi liquid behavior with a *T*^2^ relation. No apparent dependency of the linear-in-*T* resistivity, besides a rather strong change of prefactor, is found when applying displacement fields as long as the filling is tuned to the VHSs, including D ∼ 0.28 V/nm where a high-order VHS is expected. Intriguingly, such non-Fermi liquid linear-in-*T* resistivity persists even when magnetic fields break the spin-degeneracy of the VHSs at which point two linear in *T* regions emerge, for each of the split VHSs separately. This points to a mechanism of enhanced scattering at generic VHSs rather than only at high-order VHSs or by a quantum critical point during a Mott transition. Our findings provide insights into the many-body consequences arising out of VHSs, especially the non-Fermi liquid behavior found in moiré materials.

## Significance

Many quantum materials exhibit an intriguing strange metal phase, characterized by a linear in temperature resistivity. The relation of this phase to other emergent quantum many-body phases, such as superconductivity, triggers an intense debate to this date. Here, we study twisted bilayer (TB-) WSe_2_ and report on the tunability of emergent phenomena driven by the high density of states caused by vanishing slopes in the dispersion relation: so-called Van Hove singularities (VHSs). We observe that linear in temperature resistivity closely follows the positions of the VHSs independent of the presence or absence of quantum criticality and seemingly agnostic of their high-order nature. Our findings establish TB-WSe_2_ as a promising platform to investigate the relevance of VHSs for quantum materials.

The electrical resistivity of metallic states at low temperatures (*T*) exhibits *T*^2^ dependence, which follows the Landau-Fermi liquid theory where the average relaxation time for electrons on the Fermi surface is inversely proportional to *T*^2^ (1–4). There are a few marked exceptions in which the very general Fermi liquid behavior breaks down, for example, the unconventional high-*T*_c_ superconductors above *T*_c_ (5–10) and Mott insulators doped to their quantum critical points (11–13). In such systems, resistivity shows a linear *T* dependence, referred to as a strange metal (9, 10, 14, 15), a strongly correlated phenomenon that has not been yet well understood. Early considerations of van Hove singularities (VHSs) led to the idea that enhanced electron–electron or electron–phonon scattering might also led to linear in *T* resistivity (16, 17), however, at least for the electron–electron part, this scenario was not supported by a dynamical mean field analysis (18). Intriguingly, recent theories proposed that a *T*-linear behavior can also result from special, extended types of VHSs where the density of states (DOS) exhibits a power-law divergence in two-dimensional systems, known as high-order van Hove singularities (hVHSs) (19–22). Due to its pronounced divergence, an hVHS can also induce ordering instabilities and give rise to exotic correlated phenomena, including supermetal, ferromagnetism, and chiral superconductivity (20, 23–25).

High-order VHSs have been theoretically reported in various conventional systems before, like cuprate high-*T*_c_ superconductors (26, 27), Sr_3_Ru_2_O_7_ (28), and bilayer graphene (19). However, a systematic transport experimental characterization of their properties was up to recently hindered by the lack of sufficiently controlled material platforms. With the advent of tunable moiré systems for which theory proposed the existence of hVHSs, there is a unique opportunity to investigate hVHSs, since the flat band in these systems has lower energy which enable us to access their band characteristics through transport measurements. Among the various theoretical predictions regarding moiré systems (21, 29–31), twisted homobilayer transition metal dichalcogenides (TMDs) systems have unique advantages, they feature spin-valley locking simplifying the band degeneracy to two, much simpler compared to twisted graphene systems. Moreover, in twisted homobilayer TMDs exist both conventional VHSs and high-order VHSs, with one type easily transitioning to the other by applying displacement fields, and allow for the exploration of hVHSs (29, 32, 33).

Recently, quantum critical behavior near half-filling correlated insulating state has been reported in 4.2° TB-WSe_2_ device (13). However, under such a twisted angle, theory suggests that the VHS is also expected to appear right at the half-filling position where the Mott insulator exists (33) and that a hVHS point is nearby with respect to changing the displacement field. In this case, disentangling the property of the VHSs, hVHSs, and the Mott insulator physics can be challenging, as doping a Mott insulator to the quantum critical point can also induce a *T*-linear behavior of the resistivity. In this paper, we choose smaller twisted angles, for which the hVHSs are further away from the half-filling correlated insulating state. This allows to access the unperturbed VHSs and hVHSs physics and to tune in and out of the Mott regime. Thus, our work can shed light on the mechanism driving linear in *T* resistive behavior. We use the ‘tear and stack’ method (34–36) to fabricate multiple TB-WSe_2_ devices with AA stacking order (37). This method combines prepatterned Hall bar-shaped Pt leads (38) (*SI Appendix*, Fig. S1) and a dual-gate structure to achieve a lower contact resistivity at low temperatures (39), similar to previous works (13, 40). Multiple devices show similar results. Here, we mainly focus on the 3.2° TB-WSe_2_ device.

**Fig. 1.**
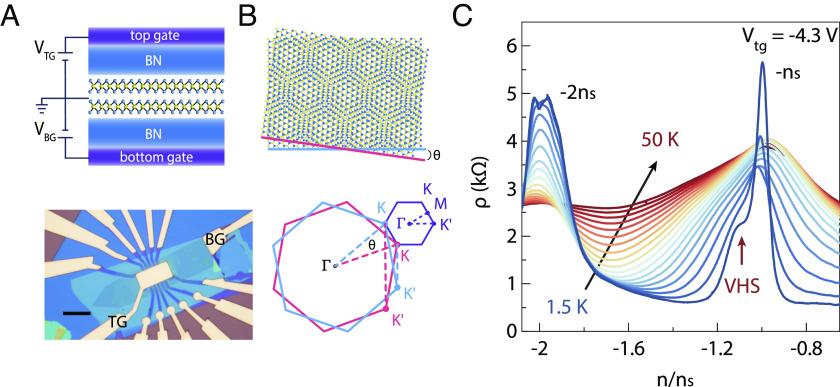
Device structure and correlated insulating state in TB-WSe_2_. (*A*) *Top* panel: The illustration of the TB-WSe_2_ device with a dual-gate structure. *Bottom* panel: The optical image of a real TB-WSe_2_ device. Black scale bar: 10 μm. “BG” and “TG” denote the back gate, and top gate respectively. (*B*) *Top* panel: real-space representation of the moiré pattern that results from a AA stacking-order between the two WSe_2_ layers. *Bottom* panel: Brillouin zones of the top (red line) and bottom (blue line) layers. The resulting band structure in the moiré Brillouin zone (purple) is also displayed. (*C*) Longitudinal resistivity ρ plotted versus the normalized carrier density n/n_s_ at a top gate voltage V_tg_ of −4.3 V for different temperatures. It demonstrates that both the half-filling and full-filling states exhibit insulating behavior. The asymmetrical part of the half-filling peak marked by a red arrow at 1.5 K can be attributed to the VHSs being slightly shifted away from the half-filling.

In Fig. 1*A*, the top panel shows an illustration of a TB-WSe_2_ device with a dual-gate structure in which we can independently tune the displacement fields D and carrier density n (36, 40). The corresponding optical image of a real TB-WSe_2_ device is displayed in the bottom panel. Schematic illustrations of the moiré superlattice in real space (*Top* panel) and reciprocal space (*Bottom* panel) are shown in Fig. 1*B*. We also calculate the band structure of TB-WSe_2_ with a twist angle of 3.15° (*SI Appendix*, Fig. S2). Fig. 1*C* shows the longitudinal resistivity ρ as a function of the normalized carrier density n/n_s_ at a top gate voltage V_tg_ of −4.3 V and various temperatures. Here, n_s_ denotes populate one hole in each moiré unit cell. We can observe two prominent peaks, corresponding to a half-filling correlated state and a full-filling state, respectively. Notably, both peak values decrease with increasing temperature, confirming their insulating nature. Furthermore, we also observe that the half-filling peak exhibits asymmetric behavior at 1.5 K, which can be seen as the VHSs deviating slightly from half-filling position.

**Fig. 2.**
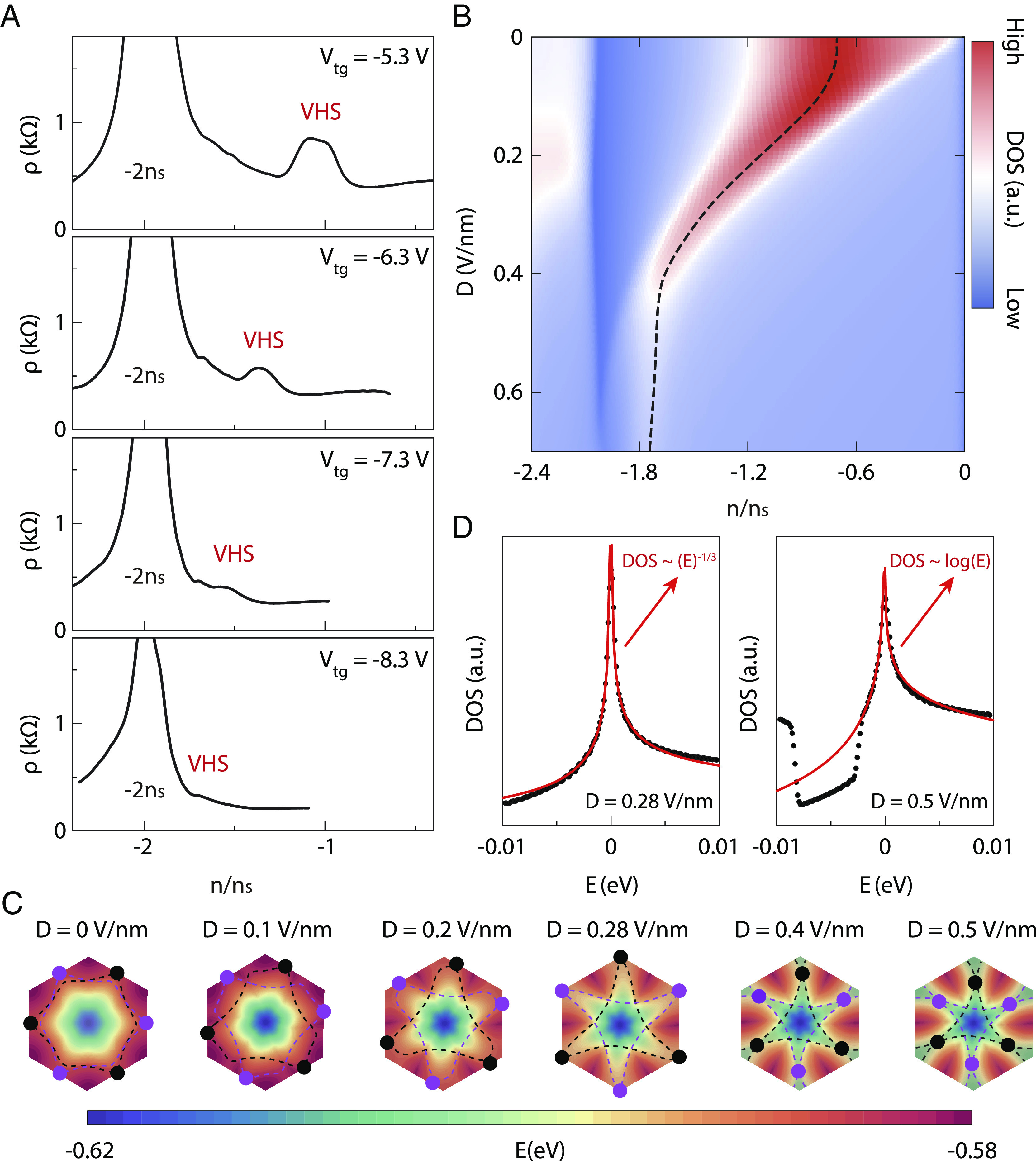
Tunable van Hove singularity and the Lifshitz transition in band structure induced by displacement fields in TB-WSe_2_. (*A*) Longitudinal resistivity *ρ* versus normalized carrier density n/n_s_ at various top gate voltages and *T* = 1.5 K. As the top gate voltage increases, the VHSs move toward the full-filling position. (*B*) The evolution of the calculated DOS with the normalized carrier density n/n_s_ and displacement field D, which provides a complete picture of the tunability of VHSs by displacement fields. The dashed black lines trace the position of the VHSs. (*C*) The calculated band structure within the first moiré Brillouin zone for 3.15° TB-WSe_2_ device at a variety of displacement fields D. The dashed purple (black) lines mark the Fermi contour at the van Hove energy in the K (K′) valley. The purple (black) points mark VHSs in the K (K′) valley. (*D*) DOS plotted against energy E at displacement fields of 0.28 V/nm (*Left* panel) and 0.5 V/nm (*Right* panel), respectively. The red curves represent fitting curves. In the *Left* panel of *D*, at D = 0.28 V/nm, the high-order VHSs form, resulting in a power-law divergence with E. Conversely, in the *Right* panel of *D*, at D = 0.5 V/nm, the high-order VHSs revert back into conventional VHSs, exhibiting a logarithmic divergence with E.

To investigate the tunability of the VHSs in TB-WSe_2_, we measure the longitudinal resistivity ρ plotted against the normalized carrier density n/n_s_ at V_tg_ of −5.3 V, −6.3 V, −7.3 V, and −8.3 V, respectively, as shown in Fig. 2*A*. By applying a displacement field, we successfully achieve the tuning of the VHSs within a wider range of carrier densities than that in previous work (13, 40) which can be due to a smaller twisted angle here. Moreover, we notice that the peak value of the VHSs sharply weakens with applying larger top gate values. Similar phenomena have also been observed in other TB-WSe_2_ devices (*SI Appendix*, Fig. S3). Fig. 2*B* illustrates the DOS calculated by the Tight-Binding method as a function of the normalized carrier density n/n_s_ and displacement field D, where the dashed black line marks the DOS peak positions. When applying a displacement field D, the DOS peak position can be effectively tuned, moving from below the half-filling to above the half-filling, and then further toward the full filling. Moreover, we notice that the strength of the DOS peak decreases monotonically across the entire range of displacement fields. To compare with our experimental results, we convert the top gate values in Fig. 2*A* into displacement fields using the formula in previous works (13, 40). We determine that applied displacement fields ranging from ∼0.24 V/nm to ∼0.5 V/nm in this 3.2° TB-WSe_2_ device. Within this range, the changes in resistivity peaks of the VHSs are consistent with the changes observed in the DOS peak in the calculated phase diagram.

Furthermore, we calculate the band structure for this TB-WSe_2_ device within the first moiré Brillouin zone at various displacement fields, as shown in Fig. 2*C*. The purple (black) dashed lines within each moiré Brillouin zone mark the Fermi surface at the van Hove energy in the K (K′) valley, respectively. By applying displacement fields D, the VHSs can be evidently moved within the moiré Brillouin zone. In particular, when D reaches a critical value Dc of about 0.28 V/nm, three conventional VHSs can merge into a high-order VHSs (21, 29, 32, 33, 41, 42) at the corners of the Brillouin zone marked by purple (black) points in the K (K′) valley. In this case, the corresponding density of states exhibits a power-law divergence with energy, as shown in the *Left* panel of Fig. 2*D*. As D further increases, i.e., when D equals 0.5 V/nm, the high-order VHSs move toward the center of the Brillouin zone and revert back to conventional VHSs, in which the density of states exhibits a logarithmic divergence, as illustrated in the *Right* panel of Fig. 2*D*.

**Fig. 3.**
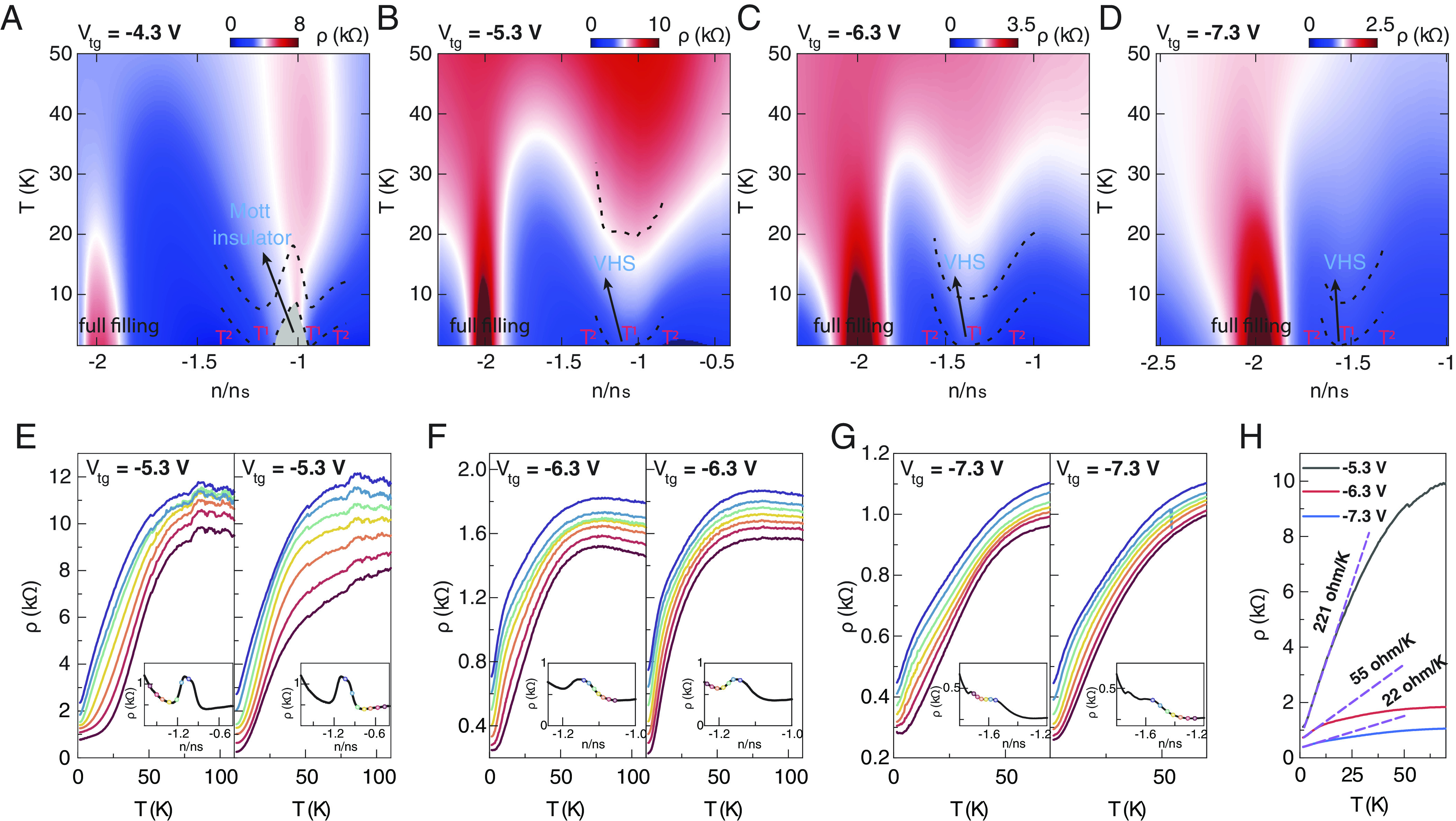
Non-Fermi liquid behavior in TB-WSe_2_. (*A*–*D*) Longitudinal resistivity ρ as a function of temperature T and normalized carrier density n/n_s_ at a series of top gate voltage of −4.3 V, −5.3 V, −6.3 V and −7.3 V respectively. Black dashed lines separate regions of Fermi liquid *T*^2^ and non-Fermi liquid T resistivity. (*E*–*G*) *ρ* plot against *T* curves at V_tg_ of −5.3 V, −6.3 V and −7.3 V respectively. Each curve corresponds to a point in the inset figure with the same color. These curves are offset for clarity. As we approach the VHSs from higher (lower) doping, the temperature dependence of *ρ* changes from a Fermi-liquid behavior with a *T*^2^ relation to a *T*-linear behavior. (*H*) The temperature dependence of ρ for a filling at the VHSs for three different top gate voltages of −5.3 V, −6.3 V, and −7.3 V, respectively. The purple dashed lines represent the corresponding linear fits at low *T*. The fitting ranges are from 1.5 K to approximately 19 K, 10 K and 7 K for V_tg_ values of −5.3 V, −6.3 V, and −7.3 V, respectively. From linear fitting, we obtained zero-temperature extrapolation values of 0.66 kΩ, 0.64 kΩ, and 0.36 kΩ, respectively, for V_tg_ values of −5.3 V, −6.3 V, and −7.3 V.

Next, we focus here on the temperature dependence of the longitudinal resistivity around the half-filling correlated insulating state and VHSs regions, to elaborate on the emergence of *T*-linear dependence in resistivity measurements. Fig. 3 *A*–*D* shows the ρ as a function of n/n_s_ and *T* at top gate voltages of −4.3 V, −5.3 V, −6.3 V, and −7.3 V, respectively, where *T* linear and *T*^2^ regions around the Mott insulator and VHSs regions are marked by black dashed lines. When a top gate voltage is set to −4.3 V, we observe that the *T*-linear regions enclosing the half-filling correlated insulating states (marked by gray shadow), which is consistent with the previous findings of ref. 13. When the top gate voltage is increased to −5.3 V, although the Mott insulator has already collapsed due to the increasing bandwidth with increased displacement fields (40), interestingly, we can still observe a *T*-linear region at low temperatures within the VHSs resistivity peak region, as shown in Fig. 3*B*. As we dope away from the VHSs, *T*^2^ regions of resistivity appear on both of its sides. Moreover, similar phenomena are also observed in Fig. 3 *C* and *D* but with a shrinking T-linear region in both carrier density and temperature ranges, which might be attributed to the weaker electron–electron or electron–phonon interaction since the bandwidth of the flat band increased further under larger displacement fields.

To clarify this change, we plot resistivity ρ versus temperature *T* for the doping range around the VHSs resistivity peak at top gate voltages of −5.3 V, −6.3 V, and −7.3 V, respectively, as shown in Fig. 3 *E*–*G*. Each curve corresponds to the point in the inset of the figure with the same color. In Fig. 3*E*, as we approach the VHSs resistivity peak from higher (*Left* panel) or lower doping (*Right* panel), Fermi-liquid behavior with a *R* ∼ *T*^2^ relation gradually transitions into a non-Fermi liquid behavior with a *T*-linear dependence. The other two plots, Fig. 3 *F* and *G*, exhibit similar tuning behavior with doping but show a slower resistivity decreasing trend with decreasing temperature at low temperatures. In this case, we also plot *ρ* of the VHSs against *T* at these three top gate voltages of −5.3 V, −6.3 V, and −7.3 V, respectively, as displayed in Fig. 3*H*, where the dashed purple lines indicate the corresponding fitting curves. We observe a prominent change in the value of the slope which is maximal ∼221 Ω/K at V_tg_ of −5.3 V and then sharply decreases to a small value. Based on the understanding of the band structure Fig. 2*C*, we deduce the sudden slope drop might be a consequence of large bandwidth changes as we tune through the hVHS condition. However, it is important to note that overall *T*-linear resistivity behavior seems to closely follow the VHS filling points, even when these filling are detuned from the Mott condition of half filling. Additionally, they prevail even when tuning into or out of the hVHS condition. This renders a mechanism for the non-Fermi liquid behavior that does not invoke quantum criticality or the exact conditions of a hVHS to be the most promising (such as VHS-enhanced phonon-scattering). Moreover, as the *T*-linear resistivity only persists at temperature less than a few tens of Kelvins in all our TB-WSe_2_ ranging from 3 to 6 degrees (Fig. 3 and *SI Appendix*, Fig. S4), the physics underlying this mechanism may differ from that of optimally doped high-Tc cuprates, where the T-linear resistivity dependence extends to relatively high temperatures (10). Similar phenomena have also been observed in other TB-WSe_2_ devices (*SI Appendix*, Fig. S4).

**Fig. 4.**
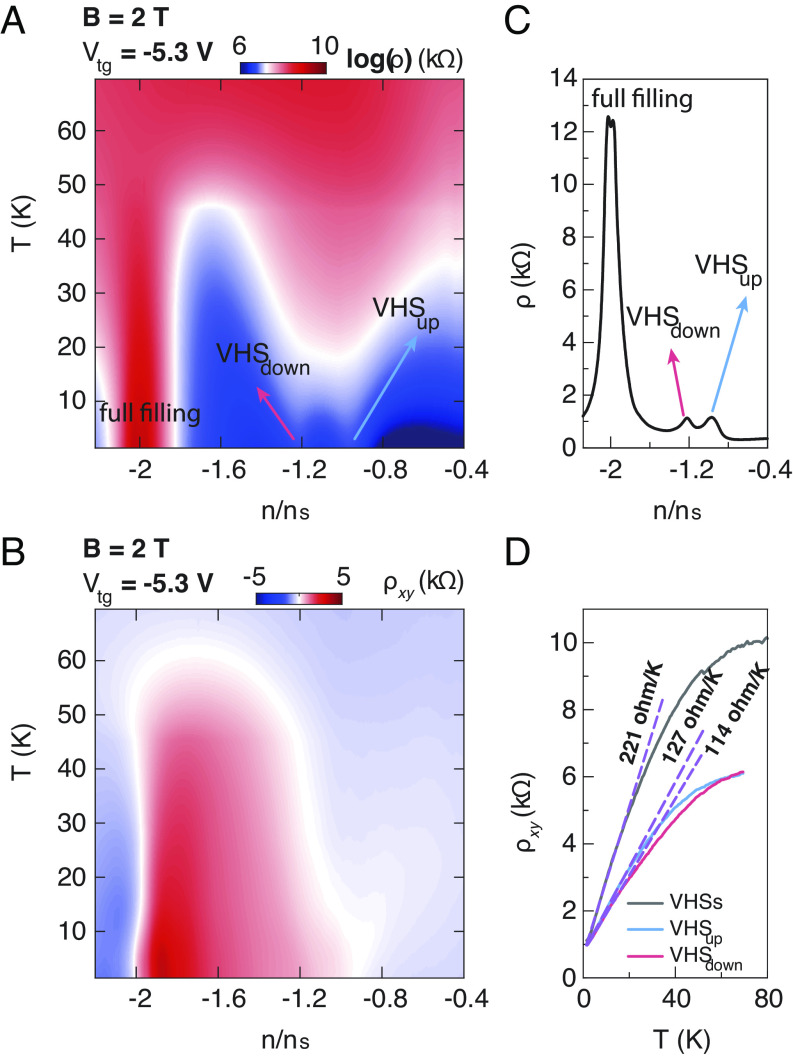
Correlation of the T-linear behavior of VHSs under a small magnetic field in TB-WSe_2_. (*A* and *B*) Longitudinal resistivity ρ and Hall resistivity ρ_xy_ as a function of temperature T and normalized carrier density n/n_s_ at a top gate voltage of −5.3 V (B = 2 T). When a magnetic field is applied, the spin degeneracy is lifted, resulting in two single VHSs denoted as VHS_up_ and VHS_down_, respectively. (*C*) A line-cut of (A) at T = 1.5 K. (*D*) ρ plotted against temperature T at a top gate voltage of −5.3 V for the VHSs, VHS_up_ and VHS_down_, respectively. The dashed purple lines represent the corresponding fitting lines.

Finally, we apply a perpendicular magnetic field to lift the two-fold spin degeneracy in TB-WSe_2_ in order to study the correlation of the T-linear behavior as the VHSs split. Fig. 4 *A* and *B* show the longitudinal resistivity ρ and Hall resistivity ρ_xy_ as a function of normalized carrier density n/n_s_ and temperature *T* at V_tg_ of −5.3 V under a magnetic field of 2 T, and a line cut of Fig. 4*A* at 1.5 K is shown in Fig. 4*C*. In Fig. 4*A*, we observe that the resistivity peak of the VHSs splits into two, which is marked as VHS_up_ and VHS_down_, respectively. Moreover, the resistivity peaks of both VHS_up_ and VHS_down_ broaden and enhance with increasing temperatures. The corresponding ρ_xy_ displayed in Fig. 4*B* exhibits two sign changes at the lowest temperature. One of the sign changes occurs at the full filling due to the charge carriers change from hole-like to electron-like when the Fermi level passes through the band gap. The other the sign change appears at the VHS_up_ position. This is because the carrier type also changes when crossing a VHS due to the sign change of the band curvature. However, when the magnetic field splits one VHS into two, the overall carrier type depends on the details of the overlap between different carrier types determined from each VHS, which could result in only one sign change in the ρ_xy_ at one of the VHSs as we observed in experiments. Similar phenomena are also observed at other V_tg_ values in this device (*SI Appendix*, Fig. S5). Further, we plot the temperature dependence of the ρ for VHS_up_ and VHS_down_, respectively, as shown in Fig. 4*D* together with the B = 0 T curve for comparison. We observe that each single VHS also exhibits a non-Fermi liquid behavior. Additionally, using the linear fitting, we notice that the slope values for both the VHS_up_ and VHS_down_ reduce by about half compared to the 0 T twofold degenerate case.

In conclusion, we demonstrate the tunability of the VHSs by applying displacement fields in multiple TB-WSe_2_ devices via transport measurements. Our band structure calculations reveal that when the displacement field reaches a critical value of ∼0.28 V/nm in 3.2° TB-WSe_2_ device, a Lifshitz transition occurs, resulting in the formation of high-order VHSs. Using displacement and magnetic fields as well as control over the filling we analyze the relevance of quantum critical points, the presence of high-order VHSs and regular VHSs for the emergence of non-Fermi liquid, linear in *T* resistivity. We find that the non-Fermi liquid behavior seems to be induced by the presence of VHSs independent of the presence or absence of quantum criticality and seems to be agnostic of their high-order nature. Furthermore, when a small magnetic field is applied to break the spin degeneracy and split the VHSs into two, each individual VHS also shows a strong *T*-linear dependence with a twofold decrease in the slope value. Our findings underline that twisted homobilayer TMDs systems are promising platform for investigating quantum criticality and regular or high-order van Hove singularity and their interplay with high tunability. Moiré materials engineering can therefore shed light on the driving physics of non-Fermi liquids which allows us to access and simulate regimes, which could be relevant to broader quantum materials research endeavors. For example, these results should be helpful to a similar debate on the origin of non-Fermi liquid behavior and the relevance of VHSs in SRO compounds, such as Sr_2_RuO_4_ (43–46).

## Supplementary Material

Appendix 01 (PDF)

## Data Availability

All study data are included in the article and/or *SI Appendix*.

## References

[1] L. Landau, On the theory of the fermi liquid. Sov. Phys. JETP **8**, 70 (1959).

[2] T. Rice, Landau fermi-liquid parameters in Na and K. Phys. Rev. **175**, 858 (1968).

[3] D. Neilson, Landau fermi liquid theory. Aust. J. Phys. **49**, 79–102 (1996).

[4] G. Baym, C. Pethick, Landau fermi-liquid theory: concepts and applications (WILEY‐VCH Verlag GmbH & Co. KGaA2008).

[5] P. W. Anderson, The Theory of Superconductivity in the High-Tc Cuprate Superconductors (1997).

[6] J. L. Tallon , Critical doping in overdoped high-tc superconductors: A quantum critical point? Phys. Status solid. **215**, 531–540 (1999).

[7] D. v. d. Marel , Quantum critical behaviour in a high-t c superconductor. Nature **425**, 271–274 (2003). 13679910 10.1038/nature01978

[8] B. Keimer, S. A. Kivelson, M. R. Norman, S. Uchida, J. Zaanen, From quantum matter to high-temperature superconductivity in copper oxides. Nature **518**, 179–186 (2015). 25673411 10.1038/nature14165

[9] C. M. Varma, Linear in temperature resistivity and associated mysteries. arXiv [Preprint] (2019). 10.48550/arXiv.1908.05686 (Accessed 15 August 2019).

[10] A. Legros , Universal t-linear resistivity and planckian dissipation in overdoped cuprates. Nat. Phys. **15**, 142–147 (2019).

[11] Y. Ōno, R. Bulla, A. Hewson, M. Potthoff, Critical behaviour near the metal-insulator transition of a doped mott insulator. Eur. Phys. J. B **22**, 283–290 (2001).

[12] T. Li , Continuous mott transition in semiconductor moire superlattices. Nature **597**, 350–354 (2021). 34526709 10.1038/s41586-021-03853-0

[13] A. Ghiotto , Quantum criticality in twisted transition metal dichalcogenides. Nature **597**, 345–349 (2021). 34526705 10.1038/s41586-021-03815-6

[14] Y. Cao , Strange metal in magic-angle graphene with near planckian dissipation. Phys. Rev. Lett. **124**, 076801 (2020). 32142336 10.1103/PhysRevLett.124.076801

[15] R. L. Greene, P. R. Mandal, N. R. Poniatowski, T. Sarkar, The strange metal state of the electron-doped cuprates. Ann. Rev. Condens. Matter Phys. **11**, 213 (2020).

[16] P. A. Lee, N. Read, Why is tc of the oxide superconductors so low? Phys. Rev. Lett. **58**, 2691–2694 (1987). 10034820 10.1103/PhysRevLett.58.2691

[17] P. C. Pattnaik, C. L. Kane, D. M. Newns, C. C. Tsuei, Evidence for the van hove scenario in high-temperature superconductivity from quasiparticle-lifetime broadening. Phys. Rev. B **45**, 5714–5717 (1992). 10.1103/physrevb.45.5714 10000299

[18] R. Žitko, J. Bonča, T. Pruschke, Van hove singularities in the paramagnetic phase of the hubbard model: DMFT study. Phys. Rev. B **80**, 245112 (2009).

[19] A. Shtyk, G. Goldstein, C. Chamon, Electrons at the monkey saddle: A multicritical Lifshitz point. Phys Rev B **95**, 035137 (2017).

[20] H. Isobe, L. Fu, Supermetal. Phys. Rev. Res. **1**, 033206 (2019).

[21] N. F. Yuan, H. Isobe, L. Fu, Magic of high-order van hove singularity. Nat. Commun. **10**, 5769 (2019). 31852901 10.1038/s41467-019-13670-9 PMC6920381

[22] N. F. Yuan, L. Fu, Classification of critical points in energy bands based on topology, scaling, and symmetry. Phys Rev. B **101**, 125120 (2020).

[23] P. Igoshev, A. Katanin, Ferromagnetic instability in itinerant fcc lattice electron systems with higher-order van hove singularities: Functional renormalization group study. Phys. Rev. B **107**, 115105 (2023).

[24] L. Classen, A. V. Chubukov, C. Honerkamp, M. M. Scherer, Competing orders at higher-order van hove points. Phys. Rev. B **102**, 125141 (2020).

[25] Y. P. Lin, R. M. Nandkishore, Parquet renormalization group analysis of weak-coupling instabilities with multiple high-order van hove points inside the brillouin zone. Phys Rev. B **102**, 245122 (2020).

[26] N. Doiron-Leyraud , Pseudogap phase of cuprate superconductors confined by fermi surface topology. Nat. Commun. **8**, 2044 (2017). 29229909 10.1038/s41467-017-02122-x PMC5725553

[27] R. S. Markiewicz, B. Singh, C. Lane, A. Bansil, High-order van hove singularities in cuprates and related high-tc superconductors. arXiv [Preprint] (2021). 10.48550/arXiv.2105.04546 (Accessed 10 May 2021).

[28] D. V. Efremov , Multicritical fermi surface topological transitions. Phys. Rev. Lett. **123**, 207202 (2019). 31809068 10.1103/PhysRevLett.123.207202

[29] Y. T. Hsu, F. Wu, S. D. Sarma, Spin-valley locked instabilities in moire transition metal dichalcogenides with conventional and higher-order van hove singularities. Phys. Rev. B **104**, 195134 (2021).

[30] B. Liu , Higher-order band topology in twisted moire superlattice. Phys. Rev. Lett. **126**, 066401 (2021). 33635687 10.1103/PhysRevLett.126.066401

[31] D. Guerci, P. Simon, C. Mora, Higher-order van hove singularity in magic-angle twisted trilayer graphene. Phys. Rev. Res. **4**, L012013 (2022).

[32] H. Pan, F. Wu, S. D. Sarma, Band topology, hubbard model, heisenberg model, and dzyaloshinskii-moriya interaction in twisted bilayer wse2. Phys. Rev. Res. **2**, 033087 (2020).

[33] J. Zang, J. Wang, J. Cano, A. Georges, A. J. Millis, Dynamical mean-field theory of moiré bilayer transition metal dichalcogenides: Phase diagram, resistivity, and quantum criticality. Phys. Rev. X **12**, 021064 (2022).

[34] L. Wang , One-dimensional electrical contact to a two-dimensional material. Science **342**, 614–617 (2013). 24179223 10.1126/science.1244358

[35] K. Kim , van der Waals heterostructures with high accuracy rotational alignment. Nano Letters **16**, 1989–1995 (2016). 26859527 10.1021/acs.nanolett.5b05263

[36] Y. Cao , Correlated insulator behaviour at half-filling in magic-angle graphene superlattices. Nature **556**, 80–84 (2018). 29512654 10.1038/nature26154

[37] L. Xian , Realization of nearly dispersionless bands with strong orbital anisotropy from destructive interference in twisted bilayer mos2. Nat. Commun. **12**, 5644 (2021). 34561454 10.1038/s41467-021-25922-8 PMC8463715

[38] H. C. Movva , High-mobility holes in dual-gated wse2 field-effect transistors. ACS nano **9**, 10402–10410 (2015). 26343531 10.1021/acsnano.5b04611

[39] H. C. Movva , Density-dependent quantum hall states and zeeman splitting in monolayer and bilayer wse 2. Phys. Rev. Lett. **118**, 247701 (2017). 28665633 10.1103/PhysRevLett.118.247701

[40] L. Wang , Correlated electronic phases in twisted bilayer transition metal dichalcogenides. Nat. Mater. **19**, 861 (2020). 32572205 10.1038/s41563-020-0708-6

[41] J. Zang, J. Wang, J. Cano, A. J. Millis, Hartree-fock study of the moire hubbard model for twisted bilayer transition metal dichalcogenides. Phys. Rev. B **104**, 075150 (2021).

[42] Z. Bi, L. Fu, Excitonic density wave and spin-valley superfluid in bilayer transition metal dichalcogenide. Nat. Commun. **12**, 642 (2021). 33510138 10.1038/s41467-020-20802-z PMC7843647

[43] A. Tyler, A. Mackenzie, S. NishiZaki, Y. Maeno, High-temperature resistivity of sr 2 ruo 4: Bad metallic transport in a good metal. Phys. Rev. B **58**, R10107 (1998).

[44] A. Husain , Coexisting fermi liquid and strange metal phenomena in sr2ruo4. arXiv [Preprint] (2020). https://arxiv.org/abs/2007.06670v1 (Accessed 3 July 2020).

[45] A. Hunter , The fate of quasiparticles at high-temperature. arXiv [Preprint] (2023). 10.48550/arXiv.2308.02313 (Accessed 4 August 2023).

[46] J. B. Hauck, S. Beck, D. M. Kennes, A. Georges, O. Gingras, Competition between d-wave superconductivity and magnetism in uniaxially strained sr2ruo4. arXiv [Preprint] (2023). 10.48550/arXiv.2307.10006 (Accessed 19 July 2023).

