# Supplementary material for: Linear resistivity at van Hove singularities in twisted bilayer WSe2
Source: Proc Natl Acad Sci U S A. 2024 Apr 9;121(16):e2321665121. doi: 10.1073/pnas.2321665121 (PMC11032435; doi:10.1073/pnas.2321665121)
Supplement: Supplementary file 1 — Appendix 01 (PDF) [file pnas.2321665121.sapp.pdf]

Supporting Information for

Linear resistivity at van Hove singularities in twisted bilayer WSe_2

LingNan Wei, Qiaoling Xu, YangChen He, Qingxin Li. Yan Huang, Wang Zhu, Kenji Watanabe. Takashi Taniguchi, Martin Claassen, Daniel A. Rhodes, Dante M. Kennes, Lede Xian, Angel Rubio and Lei Wang

Dante M. Kennes, Lede Xian, Angel Rubio and Lei Wang.
E-mail: dante.kennes@mpsd.mpg.de; xianlede@sslab.org.cn; angel.rubio@mpsd.mpg.de; leiwang@nju.edu.cn

This PDF file includes:

Supporting text
Figs. S1 to S5
SI References

Supporting Information Text

Methods. All of the flakes, including single layer WSe_2, h-BN, and few-layer graphite, used in this paper were obtained by mechanical exfoliation. The overall fabrication process is the same as described in our previous work (1, 2). Briefly, we first prepare a h-BN/Graphite stamp to place Hall-bar-shaped pre-patterned leads with Cr/Pt (2 nm/20 nm) evaporated on it as shown in the Fig. S1. Next, the twisted bilayer WSe_2 part is fabricated using the 'tear and pick-up' method (3, 4). Finally, the h-BN/TB-WSe_2 stamp is placed onto the pre-patterned leads. Additionally, the method used to determine the twist angles of the measured devices is the same as described in our previous work (1, 2).

First-principles calculation. The electronic properties calculations for twisted bilayer WSe_2 were performed using density functional theory with the Vienna ab initio software package (VASP) (5). The Perdew-Burke-Ernzerhof (PBE) exchange-correlation functionals (6) were employed, along with Tkatchenko-Scheffler (TS) van der Waals corrections (7, 8), which have been shown to yield results consistent with experimental observations in our previous work on WSe_2-based moiré superlattices (1). An energy cutoff of 400 eV for the plane wave basis set and a Γ-centered k-mesh of $1\times1\times1$ were used for geometry optimization and electronic structure calculations. To prevent artificial interactions between periodic slab images, a vacuum thickness greater than 15 Å was applied. All atoms were fully relaxed, ensuring a residual force less than 0.01 eV $Å^{-1}$ per atom. Considering the computational cost of superlattice calculations, while the internal atomic positions were fully optimized, the lattice constant for the moiré supercell was kept fixed at a value corresponding to the optimized lattice constant of 3.30 Å for a 1×1 unit cell with 2H stacking. For all band structure calculations, the spin-orbit coupling (SOC) effect was taken into account due to the presence of heavy elements in the WSe_2 system.

Tight-Binding calculation. To speed up the calculations and obtain the density of states (DOS) with a high resolution in the momentum space, we adopt the Tight-Binding method for the calculations of DOS. The Tight-Binding calculations are based on a twisted bilayer honeycomb lattice with one orbital for each lattice site to simulate the K-valley states in WSe_2. We adopt parameters fitted to DFT results to ensure the faithful description of the top valence flat band in twisted bilayer WSe_2 (see Fig.S1 and Fig. 13 in Ref. (1)). The lattice constants are the same as those used in the DFT calculations and the interlayer separation is set to be 6.53Å. We employed a hopping term that only depends on the separation between the two sites, similar to the one used for twisted bilayer graphene (9, 10). The onsite energy difference between the two sublattices in each layer is set to be 1.23eV. The displacement field is applied by setting additional onsite energy difference between the atomic sites in the top and the bottom layers with the same parameters as we used in our previous work (1). We apply a Gaussian smearing of 2meV in the DOS calculation with the Tight-Binding method and a k-grid as large as 40x40x1 in the supercell Brillouin zone to ensure the convergence of DOS in our calculations.

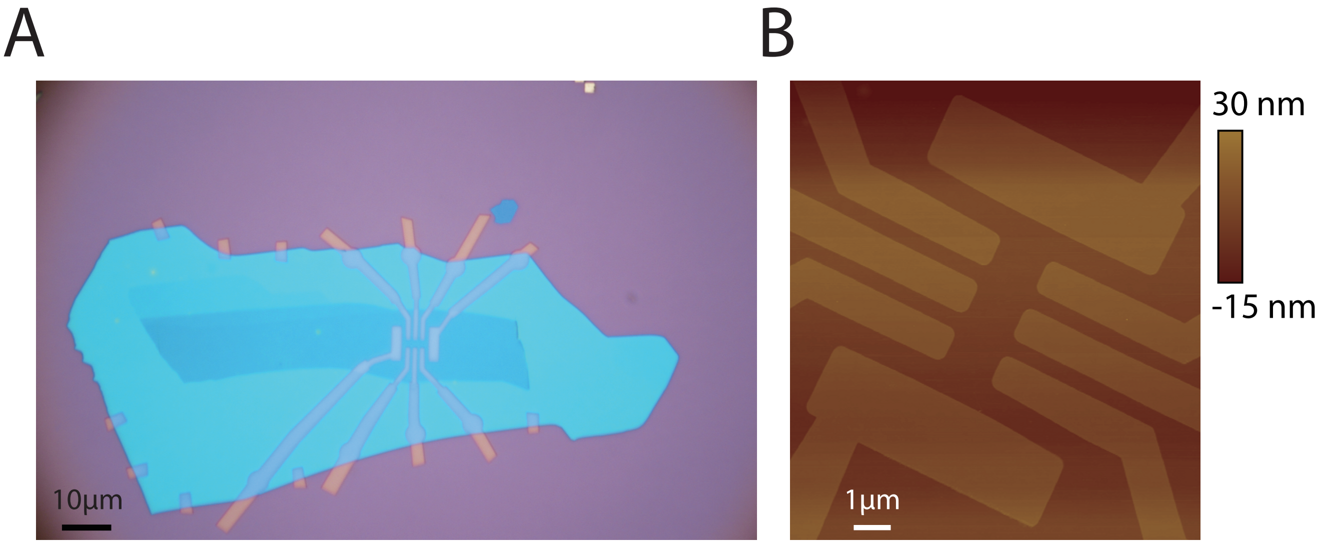

Fig. S1. Characterization of the prepattern leads. (**A**) Optical image of the prepattern leads. (**B**) AFM image of the prepattern leads in (**A**)

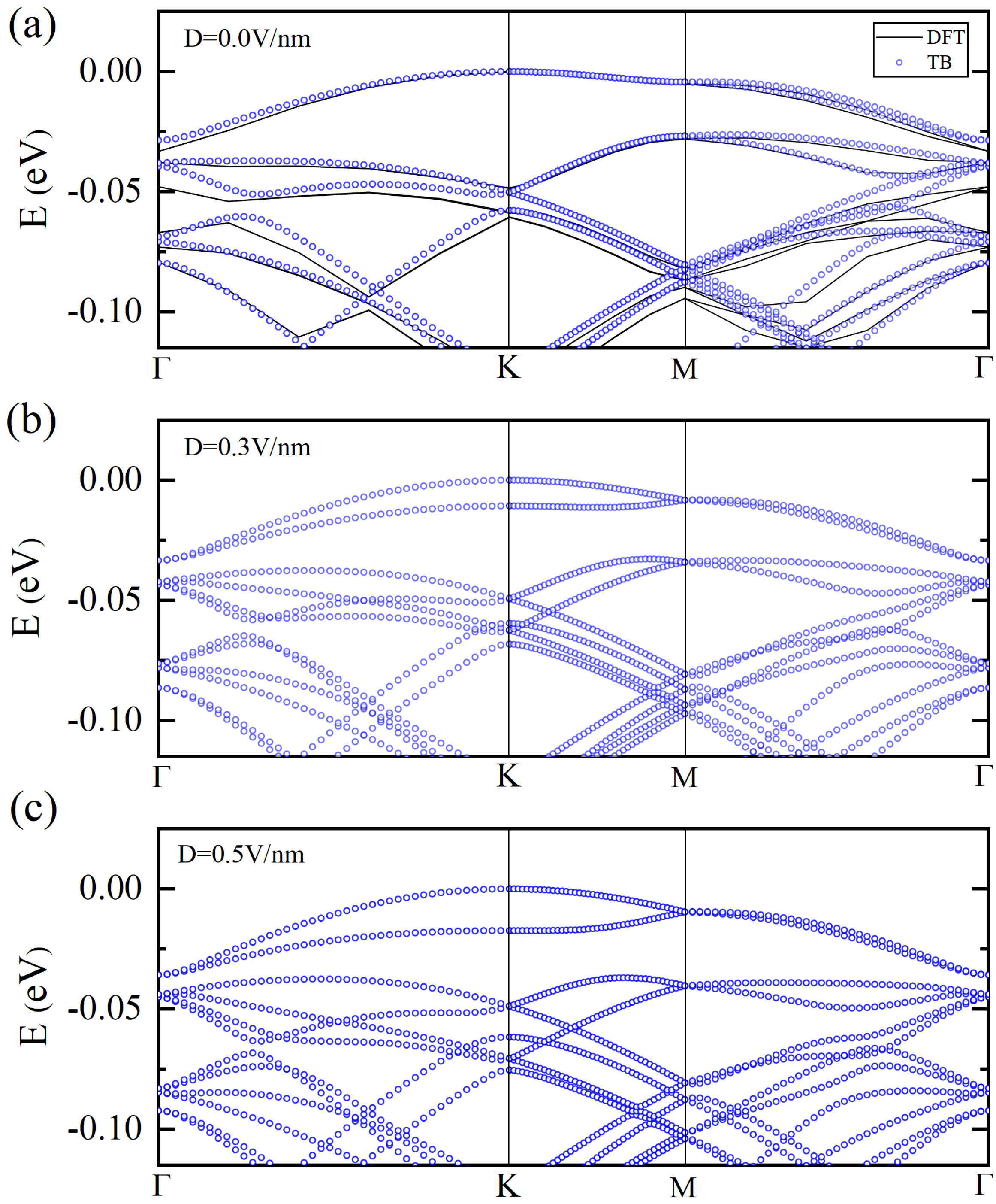

Fig. S2. Band structures of 3.15° twisted bilayer WSe_2 under different displacement fields. The results calculated by density functional theory (DFT) calculations are indicated by solid line, while those calculated by tight-binding (TB) methods are indicated by blue circles.

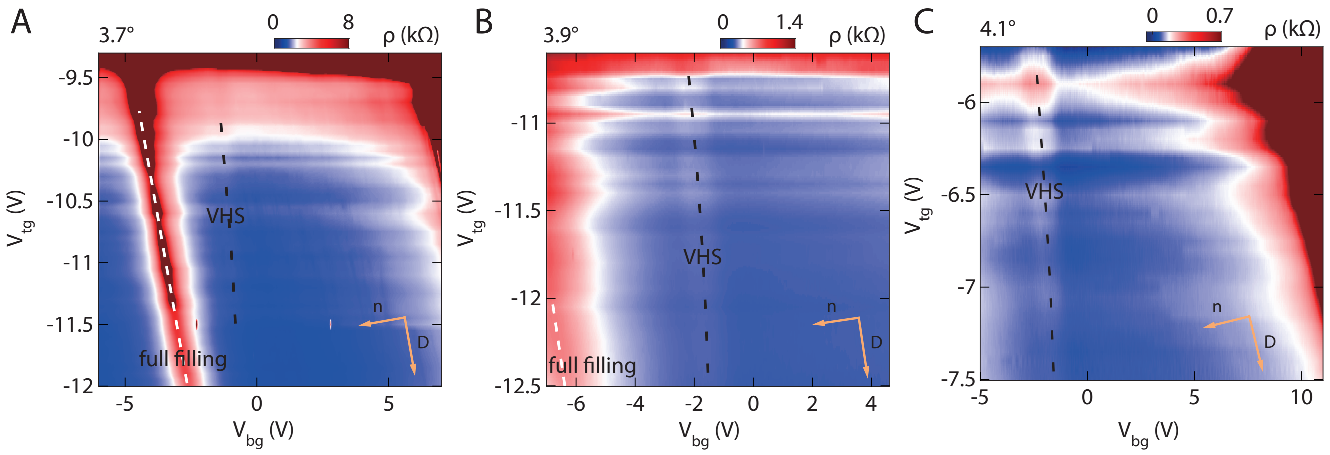

Fig. S3. Resistivity ρ as a function of top gate voltage V_{tg} and back gate voltage V_{bg} in TB-WSe$_2$ devices with different twisted angles under T = 1.5 K. The white and black dashed lines mark the full filling and VHS, respectively.

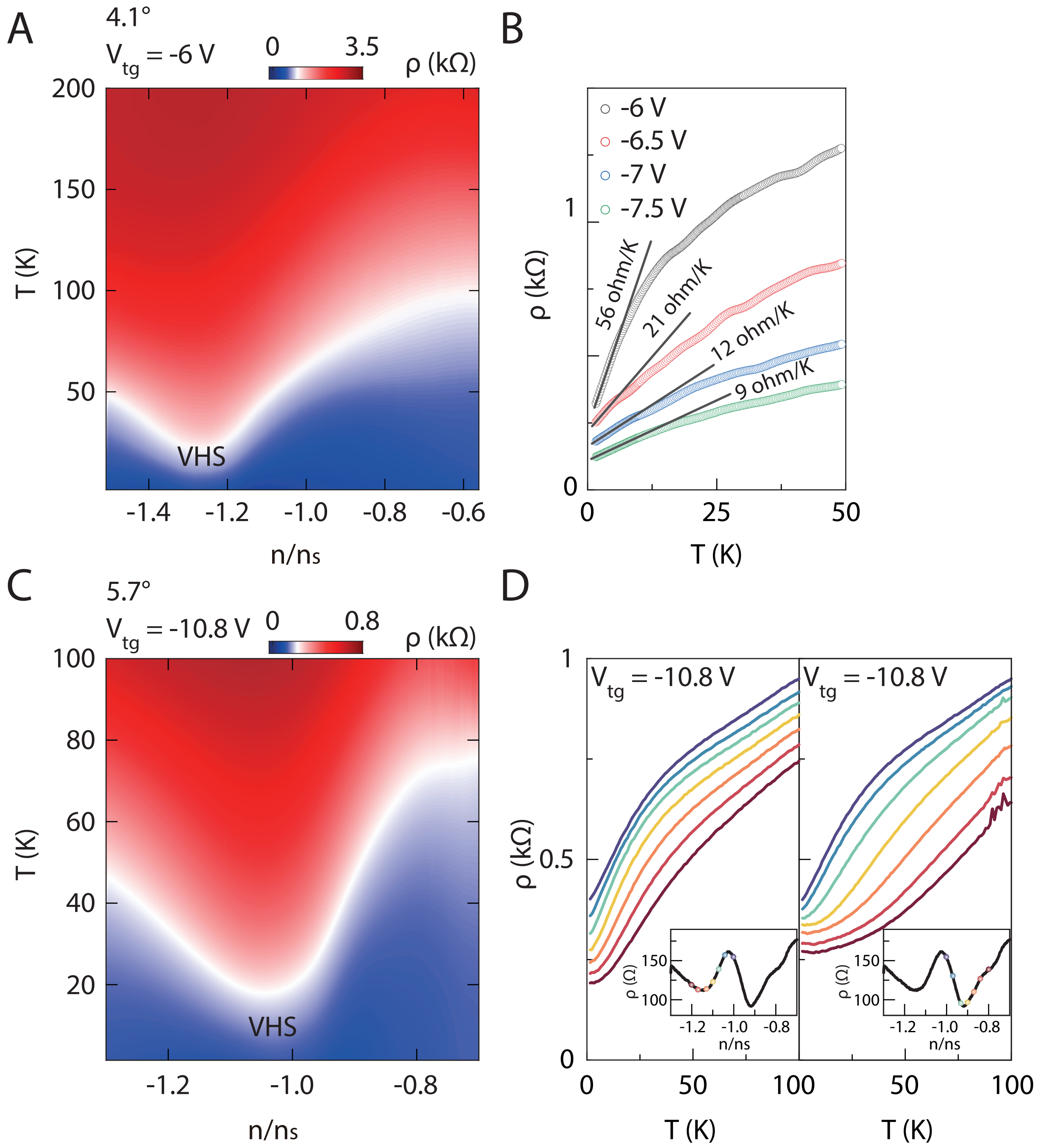

Fig. S4. Temperature dependence of the resistivity ρ in TB-WSe$_2$. (**A**) Resistivity ρ as a function of normalized carrier density n/n_s and temperature T at $V_{\rm tg}$ of -6 V in 4.1° TB-WSe$_2$ device. The resistivity of VHS shows a positive temperature dependence similar to the device in the main text. (**B**) ρ of the VHS plot against T at different $V_{\rm tg}$ values in 4.1° TB-WSe$_2$. These curves have offset for clarity. The black lines are fitting lines. (**C**) Resistivity ρ as a function of normalized carrier density n/n_s and temperature T at $V_{\rm tg}$ of -10.8 V in 5.7° TB-WSe$_2$. (**D**) ρ plot against T curves at $V_{\rm tg}$ of -10.8 V at different normalized carrier density in 5.7° TB-WSe$_2$. Each curve corresponds to a point in the inset figure with the same color. These curves are offset for clarity. As we approach the VHS from higher (lower) doping, the temperature dependence of ρ changes from a Fermi-liquid behavior with a T^2 relation to a T-linear behavior.

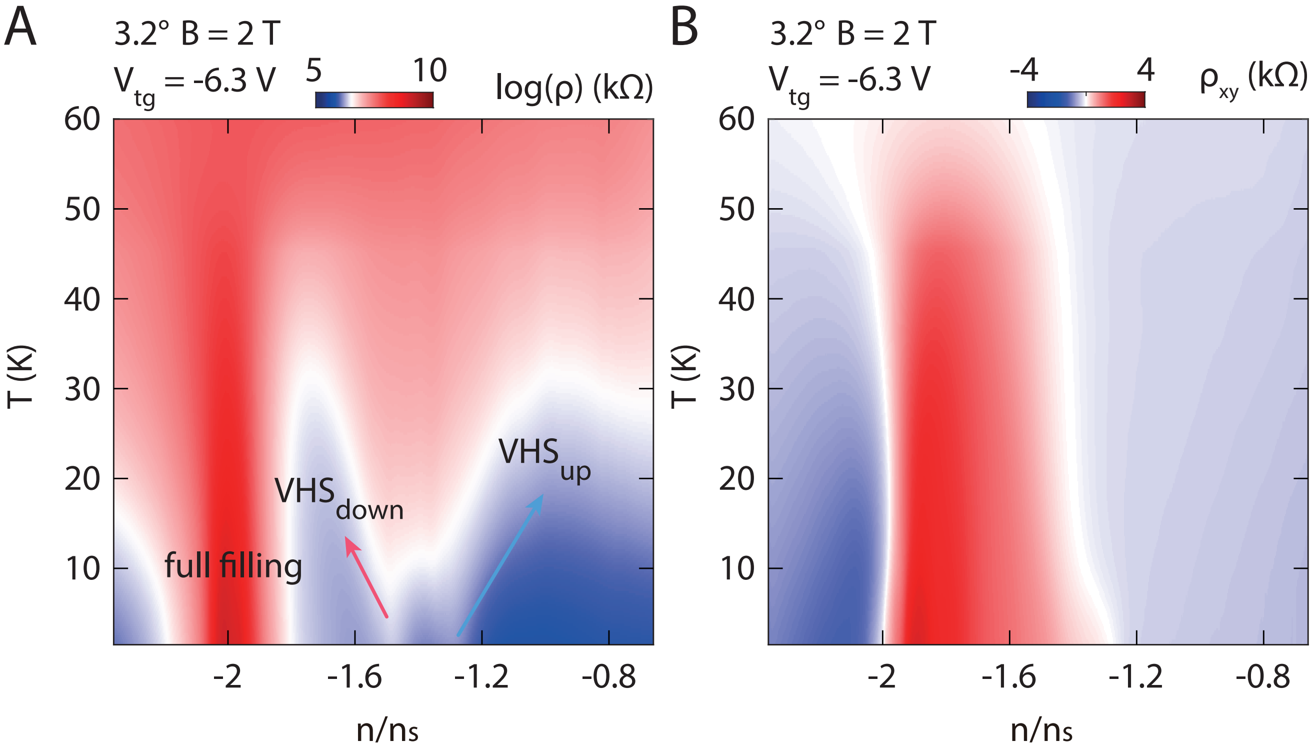

Fig. S5. Temperature dependence of the resistivity ρ and Hall resistivity ρ_{xy} in 3.2° TB-WSe$_2$ at V_{tg} of -6.3 V and B = 2 T.

References

1. L Wang, et al., Correlated electronic phases in twisted bilayer transition metal dichalcogenides. *Nat. materials* **19**, 861–866 (2020).
2. A Ghiotto, et al., Quantum criticality in twisted transition metal dichalcogenides. *Nature* **597**, 345–349 (2021).
3. L Wang, et al., One-dimensional electrical contact to a two-dimensional material. *Science* **342**, 614–617 (2013).
4. K Kim, et al., van der Waals heterostructures with high accuracy rotational alignment. *Nano Lett.* **16**, 1989–1995 (2016).
5. G Kresse, J Furthmüller, Efficient iterative schemes for ab initio total-energy calculations using a plane-wave basis set. *Phys. Rev. B* **54**, 11169 (1996).
6. PE Blöchl, Projector augmented-wave method. *Phys. Rev. B* **50**, 17953 (1994).
7. A Tkatchenko, M Scheffler, Accurate molecular van der waals interactions from ground-state electron density and free-atom reference data. *Phys. Rev. Lett.* **102**, 073005 (2009).
8. O Anatole von Lilienfeld, A Tkatchenko, Two-and three-body interatomic dispersion energy contributions to binding in molecules and solids. *The J. chemical physics* **132** (2010).
9. G Trambly de Laissardière, D Mayou, L Magaud, Localization of dirac electrons in rotated graphene bilayers. *Nano letters* **10**, 804–808 (2010).
10. GT De Laissardiere, D Mayou, L Magaud, Numerical studies of confined states in rotated bilayers of graphene. *Phys. Rev. B* **86**, 125413 (2012).
